# Socio-cultural context of adolescent sexuality and youth friendly service intervention in West Gojjam Zone, Northwest Ethiopia: a qualitative study

**DOI:** 10.1186/s12889-022-12699-8

**Published:** 2022-02-11

**Authors:** Alemtsehay Mekonnen Munea, Getu Degu Alene, Gurmesa Tura Debelew, Kerebih Asrese Sibhat

**Affiliations:** 1 School of Public Health, Bahir Dar University, Bahir Dar, Ethiopia; 2 Department of Population and Family Health, Institute of Health, Jimma University, Jimma, Ethiopia; 3 Department of social work and social development, faculty of social sciences, Bahir Dar University, Bahir Dar, Ethiopia

**Keywords:** Socio-cultural norms, Unmarried adolescent, Sexual behaviour

## Abstract

**Background:**

Recognizing that adolescents face barriers in accessing services, may feel embarrassed, face stigma on sexual matters, or have concerns about judgmental providers, youth-friendly service (YFS) has been introduced to deliver health services that meet the sexual and reproductive health (SRH) needs of young people. Evidences on the role of YFS in addressing the socio-cultural norms influence unmarried adolescent SRH behaviour are limited. Therefore, this study explore whether the socio-cultural norms influencing adolescent SRH behaviour vary between youth friendly service program and non Program areas in West Gojjam Zone, North West Ethiopia.

**Methods:**

Qualitative case study design was employed to explore the socio-cultural context of adolescent sexuality. Purposive sampling was used to identify study participants. Data were collected from 112 participants both from YFS program and non-program areas using semi-structured in-depth interviews, key informants, and focus group discussions guides. A total of 18 key informant interviews, twelve FGDs and four in-depth interviews were conducted. Participants were comprised from unmarried adolescents, parents, religious leaders, community elders, health professionals, teachers, and unmarried adolescents who experienced SRH problem. Thematic analysis was used to summarized the data.

**Results:**

The socio-cultural norms related to adolescent sexuality in both YFS program and non-program areas indicated that the community is intolerant to premarital sex, SRH service utilization (eg., contraceptive use) by unmarried adolescent; and discourage SRH communication with unmarried adolescents. According to the participants, premarital sex and SRH service use were not accepted by the community. Moreover, participants believed that, having communication on SRH issues with unmarried adolescents are equivalent to encouraging them to initiate sex, therefore, should not be practiced.

**Conclusion:**

The socio-cultural norms influencing adolescent sexual behaviour were more or less the same between settings. In both areas, the socio-cultural context discourages YFS intervention like SRH communication and service use. Also, the YFS program does not modify the socio-cultural norm affecting adolescent sexuality. Therefore, the YFS interventions strategies should give due emphasis to the socially accepted sexual norms like sexual abstinence.

## Background

Young people aged 10–24 years face various challenges during their transition to adulthood [1]. Many young people could acquire preventable health problems that might continue throughout their adult life owing to their risky sexual behaviors such as unprotected sex, multiple sexual partnerships, and transactional sex [2–4]. These behaviors predispose young people to sexually transmitted infections including HIV/AIDS, unwanted pregnancy and unsafe induced abortion [4–6]. Recognizing that adolescents face barriers in accessing services, may feel embarrassed, face stigma on sexual matters, or have concerns about judgmental providers [7–13], youth-friendly services (YFS) are introduced to deliver health services that meet the SRH needs of young people [14, 15].

The YFS approach is believed to improve adolescents’ SRH by targeting the barriers to services at the individual, social, and structural levels. At the individual level, YFS approach emphasizes increasing young peoples’ SRH knowledge, skills, and health care seeking behavior. At the social level, YFS promotes supporting community environment for adolescents’ to seek services with targeted effort of awareness activities for parents, young people, and community at large. At the structural level, YFS works by promoting national, regional, and local YFS oriented policies into the existing public health systems [16–18].

The Ethiopian government introduced YFS program in the country’s public health system in 2005. Following the inception of the program, the National Adolescent and Youth Reproductive Health Strategy (AYRHS 2006–2015) was developed. The strategy laid out the vision and objectives for increasing access and use of quality reproductive health information and services [16]. The Federal Ministry of Health also developed standards and service delivery guidelines, tools, and training curricula to assist in the implementation of YFS [19–22]. The government of Ethiopia pursued scale-up and institutionalization of YFS through intensive capacity building at all levels of the health system [16, 23]. Currently the services are given at the hospitals, health centers, universities, schools, health posts, and other community outlets including community and school outreach services [19].

YFS programs promote access to SRH services, including information and counseling on the issue, promotion of healthy sexual behaviors, and contraceptive promotion /provision regardless of age and marital status in a right-based approach [1, 24]. The rights-based approach is considered a positive approach to sexuality because it accepts sexual feelings, desire, and pleasure as essential components of young people’s sexuality [25–27]. The program encourages adolescents to abstain or delay sexual initiation until marriage or use methods that prevent unwanted pregnancy and STI. As part of the strategy, parents are encouraged to have discussions with their adolescents on sexual and reproductive health issues [16].

A comparative study between YFS program and non- program areas on SRH service utilization reported that SRH service utilization was 33.8 and 10% in the program and non- program areas [28]. A quasi experimental study on the pattern of long-acting reversible contraceptives utilization among youth also indicated that the number of new acceptors were significantly higher in the YFS program area [29]. Conversely, use of family planning was found significantly higher in the non-program area (57.4%) than the program (42.6%) [30]. Yet, these studies did not document whether the settings are providing quality YFS or not for comparison.

Other studies reported that social and cultural factors shape adolescents’ SRH experiences, decision making, and behaviors at different levels. Perceived norms about acceptability/unacceptability of adolescent sexual activity and its consequences (pregnancy, childbearing, abortion), religion and abstinence teachings about premarital sex, and limited access to quality SRH care were identified as a major factor for adolescent poor health [31, 32] Moreover, the social norms observed in different studies discourage discussion on sexual issues with unmarried adolescents [9, 33–38].

Besides, making marriage and childbearing-related decisions, individual members of the society are expected to adhere not to their personal interests, but the prevailing norms of the society [39–42]. When a person’s sexual and reproductive activities fall outside these expectations, it will likely be more difficult for that person to access information and services to meet their needs [12, 13]. For instance a qualitative study conducted in southwest Ethiopia explored that community-stigma surrounding SRH service and community condemnation of premarital sex hinder adolescent from seeking SRH information and service. This study reported that adolescents’ SRH service utilization behavior is heavily influenced by the cultural and religious norms of the community they live in [43]. Additionally, a mixed study design conducted in rural Ethiopia pointed out that sex is accepted within marriage [44]. In this regard, the role of YFS program in addressing socio-cultural barriers that prevent adolescents from having access to SRH information, care, and service are not investigated.

Thus, this study explored whether differences are existed in the socio-cultural norms related to SRH behavior of adolescents’ between YFS program and non-program areas considering the prevailing socio-cultural norms towards premarital sexual practice, parent-adolescent communication on SRH issues, and use of SRH services by unmarried adolescents. The findings may help concerned bodies to design tailored intervention to improve the SRH of unmarred adolescents.

## Methods

### Study design and Setting

A qualitative case study design was used to explore the difference on the socio-cultural norms affecting unmarred adolescent sexual behaviour. The study was carried out on September 2019 in west Gojjam Zone, Northwest Ethiopia. West Gojjam zone is divided in to 13 rural districts and 02 city administrations with 362 rural and 15 urban kebeles, respectively. The estimated population of the zone in 2016 was 2,611,925 (2,194,017 rural) [45]. In the zone there were six public primary hospitals, 104 health centers, and over 374 health posts offering basic health care operated by 51,doctors,227 public Health officers, 1016 Nurses, 293 Midwives, 266 pharmacy workers, 200 laboratory professionals, and 850 Health extension workers. In addition, there were 115 private health facilities (1 general hospital and 114 clinics of different types). Of the 104 health centers in west Gojjam Zone, 54 had YFS program [46].

The study was conducted in areas with YFS program (program area or intervention area was used interchangeably) and non-YFS program areas (non-program area or non-intervention areas was used interchangeably) to explore whether differences in the socio-cultural norms influencing adolescent sexuality. Description of the areas is presented in Table 1:

**Table 1:** Description of the study areas, west Gojjam Zone, North west Ethiopia, 2018

Characters	YFS program areas		Non-YFS program areas	
	Andasa	Brakat	Maksegnin	Kuch
Total /catchment population	37,200	45,686	23,270	37,250
Adolescent population	7440	9037	4654	7450
Kebeles (small administrative unit)	06	05	04	07
Schools	1 high school	1 high school	1 high school	1 high school
	05 elementary	08	03	05
SRH activity in the school	Yes	Yes	Yes	Yes
Peer educators	02	02	0	0
Health extension workers	15	13	09	13
Health workers in the facility	8 male	12 male	15 male	16 male
	9 female	9 female	3 female	12 female

### Participants

The participants were purposively selected from YFS program and non-program areas. Typical purposive sampling was used to select the potential research participants to be included in the focus group discussions (FGD), key informant interviews (KII) and in-depth interview (IDI). This is a procedure of selecting research participants on the basis of their relevance to the research issues. Additionally, unmarried adolescents who became pregnant following previous risky sexual behavior were included using snowball sampling. The adequacy of the sample was determined based on data saturation. A total of 112 participants included in the study. The participants comprised of different groups who were relatively knowledgeable of the prevailing socio-cultural norms regarding sexuality and governed by such norms. Accordingly, 31 unmarried adolescents (male and female) age ranging from 15 to 19 years, 30 parents who had unmarried adolescents, 18key informants comprised of religious leaders, community elders, health professionals and teachers participated in the study. In addition, four unmarried adolescents who experienced reproductive health problems (pregnancy) were included in the study.

### Data Collection tool and procedure

To collect the qualitative data, FGD, KII and IDI guides were prepared. Data were collected by five qualitative data collectors (three male and two female) with social science background and familiar to the area and experience in conducting qualitative data collection. The qualitative data collection guides were pre-tested and issues that might be encountered in the main data collection process were discussed before the actual data collection. The main data collection process was started by explaining the purpose of the study, read the consent form to each respondent and asked them to participate in the study. Once their consents were obtained, an interview date and place of interview were arranged in advance with each respondent. Those respondents who were ready to be interviewed on the first contact were interviewed on the same day of first contact. All interviews were conducted separately at respondent’s convenient hour and place. All FGDs were held in schools compounds during weekends, when the classrooms were free. The data collection from males were facilitated by trained same gender moderator and note-taker, while the focus group discussions and IDI for females were facilitated by the principal investigator and trained female note-taker. The discussions were held in Amharic (the local language) which is spoken by all participants, and they were also audio-recorded. On average, the informant interviews lasted for about 40 min while the FGD lasts about 2 h. The data were conducted to the point of saturation.

A total of 18 key informant interviews, twelve FGDs and four in-depth interviews were conducted. Of the twelve FGDs, six were conducted in the YFS program areas with unmarried female adolescents (two FGDs), unmarried male adolescent (one FGD), male parents (one FGD) and female parents (two FGDs). In a same way, the other six FGDs were conducted in the non-program areas. Additionally, in both settings eighteen KII with religious leaders, health professionals and teachers were conducted. Moreover, four IDI (two from the program and two from the non-program areas) were conducted with unmarried adolescents who had previous experience of SRH problems.

### Data quality assurance (trustworthiness)

In this study, the constructs of credibility, transferability, dependability, and confirmability were enhanced [47, 48]. To ensure credibility, different activities, including prolonged engagement, triangulation, iterative questioning, member checking, and peer debriefing were made. The principal investigator and data collectors were familiar with the cultural and social backgrounds of the study participants. Different groups of participants including families (fathers, mothers), adolescents (males, females, unmarried, married, in school, out of school, cases with the previous history of risky sexual behavior), elders, community leaders, religious leaders, health professionals and teachers in the two settings were included to have triangulated data. Data were also collected using FGD and IDI. Member checking sessions were organized to present the preliminary findings of the data. These sessions helped to confirm whether the researcher accurately understood the informant’s/discussants point.

As to transferability, a thick description was used to show that the research findings can be applied to other contexts, circumstances, and situations. This included the number of participants, where the participants are from, the number of participants involved in the fieldwork, the data collection methods that were employed, the number of activities, and the length of the data collection sessions. Lincoln and Guba stress the close ties between credibility and dependability, arguing that, in practice, a demonstration of the former goes some distance in ensuring the latter [47]. Dependability was achieved through the use of overlapping methods, such as the focus group and individual interview. Furthermore, the use of a tape recorder, careful probing, interviewing up to data saturation, and considering the difference between individuals were activities done to ensure dependability. In this study, triangulation was used to reduce the effect of investigator bias (Confirmability).

### Data Analysis

All interviews were transcribed verbatim into Amharic (local language) and translated into English and were analyzed by using thematic analysis approach. The transcriptions were read several times to understand contents and contexts. This was followed by extraction of meaning units from the transcripts. The meaning units were condensed by shortening the original text while maintaining the central meaning. The condensed versions were later assigned codes, which were grouped into similar categories (Table 2). The Principal Investigator (PI) cross-referenced between Amharic (local language) and English transcripts to ensure that the meaning units, codes and categories of the two languages were similar. This analysis used already designed coding schemes (anticipated codes/priori codes) which had been developed from the question guide. After the coding process had been completed, searches were carried out which involved thoroughly reading the individual codes for emerging patterns.

**Table 2:** The prior codes and categories from the interview involving

Category	Code	Descriptions
Socially accepted definition of adolescent	Physical Social Behavioral	-physical change (height, weight, voice change) -marriage as a demarcation as an adult - expected to participate in the social events -behavioral change (aggressive, argue with parents)
Socially accepted behaviour during adolescence	-non SRH related -SRH related	-respectful, limited movement, not argue with parents -do not stand/talk with opposite sex, not started sex
Social norm related to premarital sex	-Religious stance -Socio-cultural stance -Gender stance	-considered as a sin, violating the Lord’s wishes, disobedience of religious teachings - embracing, out of the norm, taboo, poor parenting -strict in girls, huge consequences, affect future personal development
Socially accepted SRH service use by unmarried adolescents	-contraceptive use -HIV testing	- contraceptive use is a sin, violating the Lord’s wishes - HIV testing before marriage is encouraged
SRH information to be communicated to unmarried adolescents	-protective aspect of sexuality -dangers aspect of risk sexual behaviour	- abstaining from sex -STIs, HIV and teenage pregnancy
Barriers of communication on matters related to SRH	- misconception -social norms -Age	-fear that it will initiate sex -unhelpful norms, shame, lack of SRH knowledge -too young to be told

### Findings

#### Background characteristics of informants

A total of 112 informants participated in the qualitative study. Of this, 46 and 44 were unmarried adolescents and parents respectively. Ten religious leaders and community elders (five from each) were also participated as key informants. Additionally four (two in each setting) unmarried adolescents who experienced SRH problem were included in the study. Majority of the participants were orthodox Christian and literate (Table 3).

**Table 3:** Socio-demographic characteristics of informants participated in the study, west Gojjam zone, Northwest Ethiopia, 2018

Characters	Description	YFS program		Total
		Program area	Non-program area	
Background characteristics of unmarried adolescent participated in the FGD(*n* = 46)				
Sex	Male	8	7	15
	Female	15	16	31
Age	Mean age (SD)	17.8 (1.6)	17.8 (1.3)	17.8 (1.4)
Current schooling status	In-school	13	15	28
	Out-of school	10	8	18
Religion	Orthodox	21	23	44
	Muslim	2	0	2
Background characteristics of parents participated in the FGD(*n* = 44)				
Sex	Male	7	8	15
	Female	14	15	29
Age	Mean age	54 (7.2)	50.6 (5.8)	52.5 (7.7)
Educational status	Illiterate	8	10	18
	Write and read	10	8	18
	Literate	3	5	9
Religion	Orthodox	20	22	42
	Muslim	1	1	2
Marital status	Married	17	20	37
	Widowed/divorced	4	3	7
Occupation	Farmer	16	20	36
	Employed	3	0	3
	Daily laborer	2	3	5
Characteristics of religious leaders (5), community elders (5), health workers (6) and teachers (2) participated in the Key informant interview (*n* = 18)				
Religious leaders	Orthodox	1	2	3
	Muslim	1	1	2
	Mean age	66	76	71
Community elders	Male	2	1	3
	Female	1	1	2
	Mean age	74	72	73
Characteristics of health workers (6) and teachers (2)				
Sex	Male	2	3	5
	Female	2	1	3
Age	Mean Age	30 (2.8)	27 (2.8)	27.6 (3.7)
Education	Diploma	1	2	3
	Degree	3	2	5
Work experience	< 5 years	1	2	3
	> = 5 years	3	2	5
Adolescent experienced SRH problems		2	2	4

### Conceptualization of adolescence

Adolescence is conceptualized in a similar way among informants. Discussants explained that the onset of adolescence is marked by physical and behavioral changes around the age of 15/16 years and ends when he/she started showing adult behavior at marriage. Girls often marry between the ages of 16–17 years while boys marry about 18 years and above. Once married, adolescents are considered adult members of the community and participate in different social events, such as monthly and annual festivities.

Adolescent discussants argued that marriage by itself does not necessarily indicate a passage to adulthood. In some localities, there are contexts where children marry below age 10 when only the marital ceremony might be celebrated. The couples may live with their biological parents till they reach the expected age to start married life. For these discussants, the onset of adolescence is around 15/16 years and ended when youth are married and start married life.

Both discussants (parent, community elder, and adolescents) mentioned that physical and behavioral changes are observed in both males and females during the adolescence period.

Parents and community elder discussants stated that males become masculine, grow hair in the face, and their voice becomes deepen during adolescence. Girls show breast development, growth spurt, and fat accumulation. Regarding behavioral characteristics, discussants shared that both males and females show moody, turmoil, aggressive, disobedient behavior, and desire for opposite sex during adolescence stages of development. Discussants also maintained that desire to be independent and risk-taking behaviors are often observed among males during adolescence.

### Social norm related to premarital sex

In this study, social norm related to premarital sex was more or less the same in both settings. Premarital sex is not accepted in the study settings and considered as a sin by religion. As it is clear in the following excerpts from religious leaders from the program area, premarital sex is a sin that led to punishments from the lord.

“… sex following marriage is a sacred activity. … premarital sex negates this wish. It is a sin human beings exercised violating the lord’s wishes that led to punishment from the lord such as incurable diseases.” (68-year priest from program area, Pp4).

The narration illustrates that sexual practice is allowed following marriage. Premarital sex dusts the individuals physically and morally, and has lasting consequences. Parents who participated in the discussion in both settings also stated that premarital sex is a taboo in their communities. Parents explained that the practice of premarital sex by adolescents is a sign of poor parenting and disobedience of religious teachings as follows:

“We learned that being a virgin is a sacred status for individuals. Religious leaders always reiterate this idea for the followers. They often remind us to nurture our children to grow within this belief. … premarital sex is considered as a poor follow-up from parents as well as disobedience of adolescents to their religion”. (53-year mother from non-program area, Pn2).

Parents also shared that though premarital sex is taboo in their community, a few adolescents might practice it secretly. When the event is disclosed, it might be a discussion agenda in the community. Parents maintained that:

“… especially for girls, having premarital sex is very disgraceful for herself and her family. Our culture is intolerant of such practices. We learned such practice from our parents, and we are teaching our children to avoid such practice”. (48-year mother from program area, Pp4).

The excerpt revealed that though premarital sex is forbidden for both sexes, it is very strict when it comes to girls. Premarital sex is shameful not only for the individuals who practiced it but also for the family. This thinking is intergenerational and maintained by culture.

In consonant with parents’ view, adolescent discussants in the two settings explained that premarital sex is considered as a sinful activity the society condemned most.

Both male and female adolescents shared that they are often advised to abstain from such actions that violate the Lord’s good wish for human beings. A discussant from the program area illustrated that:

“Our community discourages premarital sex for both of us [boys and girls]. It violates our Lord’s wish and it exposes adolescents to various health risks that might have lasting consequences (teenage pregnancy or unsafe abortion) … these events may interfere with girls’ education or future aspirations”. (18-year boy from program area, Pp7).

The excerpt revealed that adolescents have a similar opinion to their parents that premarital sex is not accepted by their religion. The community had a greater stake in avoiding premarital sex for girls. For girls, premarital sex has consequences that interfere in their life and development.

### Social norm related to parent-adolescent communication on SRH issues

Informants from both settings revealed that they rarely discuss sexual and reproductive health issues with their unmarried adolescents. Cultural taboos, fear that adolescents may initiate sex, and too young to be told were appealing as a common reason for not discussing SRH issues with their adolescents. The data indicated that communication topics on SRH issues seem similar between the program and non-program areas.

SRH issue is considered a very embarrassing topic to discuss. There are also taboos attached to sexuality. For instance, reproductive anatomy could not be mentioned freely by the majority of the community. And hence, the community in both settings has conservative attitudes to make meaningful communication with their children. An elder informant pointed out that

“… there are some sexual issues that I am not able to provide appropriate information for my children … . even I am worried about the terminology that should be used during communication … ., in our culture, it is taboo to call reproductive organs with its appropriate term, … . that is why we usually used synonym that may decrease the degree of embracement (laugh)”. (59-year mothert from program area, Pp3).

Moreover, discussants perceived that informing their children about sexual matters would lead them (unmarried adolescents) to engage in sex. Instead, parents usually discussed issues other than SRH. A 46-year-old man from the program area said:

“… I repeatedly advise my son to study his lesson strongly. I did not discuss reproductive health issues. If I do so, my boy may feel as I am reminding him to accomplish such unwanted activities. I also believe that it would initiate adolescent’s sexual practice”. (46-year father from program area, Pp4).

Supporting the above concern, a discussant from the non-program area stated that “… discussing about sex may lead them (children) to try it out and engage in risky sex. … this (discussion) will be like to allow them to engage in sexual activities.” 48-year father from non-program area, Pp5). In a similar vein, elder participants from the program area strongly oppose the communication, “...as a parent, one should feel ashamed to talk with their children about condom use and pregnancy prevention methods …” (68-year mother from program area, Pp8).

### Socio-cultural norm related to adolescents’ SRH service utilization

Research participants were asked about their opinion on the SRH service use of unmarried adolescents such as family planning/ condom to avoid pregnancy and STIs, STIs treatment, HIV/AIDs testing, and healthy sexuality. Also, health professionals were asked whether adolescents are using SRH services and the contexts of service provision.

Religious informants shared that the use of family planning methods is against the religious doctrine. The use of FP method is forbidden, not only for unmarried adolescents but also for married couples. Religious informants discussed that individuals who are obedient to their religion should not use family planning methods. A religious leader from the program area underlined that “we are teaching abstinence for unmarried adolescents and faithfulness for married couples. No flexibility when it comes to religion.” Informants further discussed that the use of FP methods violates the very doctrine of religiosity (procreation) and resulted in punishment from the Lord.

In the same vein, parents and community elders discussed that use of FP services should not be allowed for unmarried adolescents. They argued that this service is against the religious teachings of abstinence and the social value attached to virginity. Besides, there is a possibility that girls will be pregnant before marriage, which is very shameful in our culture, or she might be infected by STIs. A parent explained that:

“… we (parents) are advising them what is better for them …. abstinence is the only option to remain healthy for both males and females. The use of FP is against our religion and culture that values abstinence and virginity. In our community, no one advises his/her children two options- abstinence or use SRH services. We have been advising this (abstinence) and will continue in the future”. (70-year father from program area).

The excerpt revealed that parents believed that they are the observer of their children’s sexual behavior. They are working to maintain culturally accepted sexual behavior. Parents argued that the promoted SRH services violate the accepted cultural values and are not full-fledged to ensure healthy sexual behavior of adolescents.

Adolescents in both settings discussed that use of SRH service is taboo in their localities. The socio-cultural norms and attitudes regarding adolescent sexual behavior are not supportive for adolescents to access SRH services. Whenever they had a health problem, parents often decide whether the problem necessitates visiting a health facility. If there is a need to go to the health facility, parents/elder family members often accompany adolescents. They stated that those who accompanied may want to know the cause of the problem, which is embracing. Such a social environment is a barrier to adolescents’ SRH service utilization.

Some discussants hold the position that SRH services should not be used by unmarried adolescents because of two reasons. They argued that the methods included in the service violate their religious teachings (abstinence). They also stated that they doubted the efficacy of the methods used (e.g., FP for females and condom use for males) in preventing the occurrences of unwanted consequences (pregnancy or STIs). During the discussion, they presented pieces of evidence of pregnancy while taking contraceptive methods among some girls in their locality.

Furthermore, adolescents shared that when there is SRH concerns such as STIs or teen pregnancy; they often hide it and experience the worst consequences. A discussant stated that:

“As premarital sex is taboo in the locality, pregnancy/STIs keep secret. Because the prevailing norm does not accept this behavior. … there are instances that some girls had unwanted delivery while SRH services are available in the locality” 18-year girl from program area, Pp1).

The narration revealed that some adolescent girls could not use SRH services to prevent the occurrences of the problem (premarital pregnancy) due to the normative environment that serves as a barrier to services utilization. The context led to having premarital delivery. An adolescent girl who experienced such a problem illustrated it as follows:

“I had sexual intercourse while I was in grade eight. I did not expect that I would be pregnant. … I went to a nearby town for pregnancy confirmation in a private clinic. I was told that it was pregnancy and it is late for termination so that I should give birth. When my belly grows, people began to whisper at me, thus, I dropped out of school. … when my mom noticed my status, she shouted at me. All family members felt sad and ignored me. … that was unforgettable bad event in my life”. (19-year girl from non-program area, Pn1).

Regarding the SRH service use of unmarried adolescents, health professionals in the two settings were asked whether adolescents were using SRH services and the context of service provision in their health facilities. Both professionals shared a similar opinion that adolescents did not visit health facilities for prevention purposes. As to other community members, adolescents often visit the facilities when they had health problems. In the program setting, the informants explained that adolescent couples who proposed marriage often come to the health facility for HIV testing. They also reported that adolescents rarely came for termination of pregnancy.

## Discussion

This study explore whether the YFS program bring a change on the socio-cultural norms related to adolescent sexuality. We found that adolescence is highly associated with the social context of a society. The social construction of sexual maturity is more or less the same in program and non-program areas. For instance, the onset of adolescence is marked by physical changes at around 15/16 years and ends at marriage in both study areas. Girls often marry between 16–17 years and are considered an adult member of the community, once married, clearing the cultural barrier to SRH services. Similar studies in Ethiopia and other developing countries reported that, in practice, adolescence, although its onset is marked by the individual’s biological maturation, its end is determined by the social context [30, 44]. Studies in Ethiopia reported that marriage is used as a demarcation to consider a boy/girl as an adult/matured [39, 41]. Following their marriage, they are expected to give birth, participate in social events, are regarded as adults and use SRH services [41, 42]. This implies that the social environment discouraged unmarried adolescent girls to use the SRH service.

Besides, the social construction of sexual maturity, however, contradicts biological sexual maturity as marriage and childbirth are recommended after the age of 18 years. An obvious implication of this divergence in the socio-cultural and biomedical conceptualizations of adolescence is that it could make the acceptance and implementation of modern SRH intervention more challenging [12, 13, 30]. In another way direction, the YFS program does not bring a change on community belief towards sexual maturity and freedom to use SRH services.

This study also revealed that the community in the study area has culturally defined sexual behavior and norms for adolescents, and these behaviors and norms act as a social control to people’s behaviors. Also are considered as safeguards against risky sexual practices. For example, particularly for girls, being a virgin till marriage is highly encouraged, and viewed that the girl has good manners. Similarly, studies from various parts of Ethiopia revealed that abstinence is most socially acceptable norm. The social attitude attached to virginity is believed to encourage girls to stay virgins until they get married [41, 42, 44].. This implies that girls are expected to be virgins at the time of marriage; hence, premarital sexual activity is considered deviant behavior. The variations in the conceptualizations of adolescent sexuality between the socio-cultural and the biomedical stances could make the acceptance and support of modern SRH intervention more difficult. For instance, if a girl lost her virginity before marriage, she may be in difficulty to use SRH service, because the health care provider as well as herself may feel this behavior is out of the social norm [30].

This study also noted that, in both settings, premarital sex is not considered as problematic for males as it is for females. On the other side, communities in both study areas did not encourage male adolescent to use condom to prevent STIs. These cultural ambiguities may affect the SRH of unmarried boys by exposing them to unprotected sex (STIs). It also negates the idea of the modern approach (YFS) that encouraged protective sexual practice [16].

Furthermore, religion is an important governing factor in the delineation and implementation of sexual norms and values [13]. Religious based body of thought on sexuality is promoting heterosexual (started within marriage), monogamous (endorsed by marriage) and give birth soon after marriage [49]. As a result, some communities prohibit contraceptive use on the basis of religious proscriptions against both unmarried adolescent sexual activity and the use of contraceptives [13]. Similarly, participants in this study reported that premarital sex, as well as use of contraceptives is considered a sin in Ethiopian religious societies. Other studies in Ethiopia also reported that these are considered as deviant behavior- sex is only acceptable in marriage [50]; and once they marry, they should give birth soon [30]. This implies that religiously motivated reproductive norms deter the availability of contraceptive information and services for unmarried adolescents.

SRH services utilization promoted by the YFS program contradicts the coherence of the community’s normative climate. For example, safe sex practice suggested to unmarried adolescents, may be a challenge for a community to maintain or enforce norms prescribing the sequencing of sex or childbearing before marriage [13]. In this case, the community may not volunteer to adopt a strategy that affects their norm/culture.

Moreover, the social norms observed in this study seem to discourage discussion on sexual issues with unmarried adolescents. Similarly, studies in Ethiopia reported that social norms are the major barrier to discuss sexual issues with unmarried adolescents [9, 33–38].

In sum, our findings indicated that the SRH of adolescents is mainly governed by the cultural context that they live in which, in turn, has important implications on their sexual health. Such SRH related social norms challenge the implementation of YFS interventions in this community where more cultural/religious norms have considerable influence on people’s sexual behavior.

### Limitation of the study

Since respondents are not controlled in their movement between the program or non-program areas, they might have an opportunity to come into contact with YFS activities regardless of their study areas. Though the study addressed this limitation by using criteria for area selection and leaving buffer zone between settings, the results should be interpreted with these limitations in mind.

### Conclusion and recommendation

Socio-cultural norms related to unmarried adolescent sexuality were more or less the same between YFS program and non-program areas. The community is intolerant to premarital sex, SRH service utilization (eg., contraceptive use) by unmarred adolescent; and discourage SRH communication with unmarred adolescents. Such SRH related social norms may challenge the YFS interventions to bring a change on SRH in societies where more cultural/religious norms have a particularly influence on people’s health behavior. Therefore, this study would suggest to re-evaluate the YFS interventions strategies so as to give due emphasis to the socio-cultural norms surrounding adolescent’s sexual and reproductive health.

## Data Availability

The datasets analyzed during the current study are available from the corresponding author on reasonable request.
